# First Pediatric Application of Bachmann's Bundle Pacing and Left Bundle Branch Area Pacing for Bi‐Physiologic Conduction System Pacing

**DOI:** 10.1111/jce.70349

**Published:** 2026-04-21

**Authors:** Hei‐To Leung, Sabrina Tsao, Samuel Chung‐Sum Ho, Kwok‐Lap Chan, Yiu‐Fai Cheung, Sit‐Yee Kwok

**Affiliations:** ^1^ Department of Paediatrics and Adolescent Medicine Hong Kong Children's Hospital Kowloon Bay Hong Kong; ^2^ Department of Paediatrics and Adolescent Medicine, School of Clinical Medicine The University of Hong Kong Pok Fu Lam Hong Kong

**Keywords:** atrial resynchronization, Bachmann's bundle pacing, conduction system pacing, congenital complete heart block, pediatric pacing, physiological pacing

## Abstract

**Background:**

Atrial pacing at right atrial appendage causes nonphysiologic delays. Bachmann's bundle (BB) pacing preserves synchrony but lacks pediatric data.

**Case Summary:**

We report the first pediatric combined BB and left bundle branch area pacing in a 9‐year‐old boy (23.5 kg). With electrophysiologic and echocardiographic guidance, successful BB capture was achieved on a thin 2.1 mm atrial septum. Pacing significantly shortened P‐wave duration (122–> 66 ms) and reduced interatrial mechanical delay (53–> 40 ms) on speckle‐tracking echocardiography.

**Conclusion:**

This case demonstrates the acute success of BB pacing in a child. Bi‐physiologic pacing is technically feasible and safe, though long‐term outcomes warrant further evaluation.

## 1 Introduction

Physiologic pacing strategies have evolved substantially in recent years, particularly for patients requiring lifelong pacing. Conduction system pacing in the ventricle has demonstrated superior electrical synchrony and reduced risk of pacing‐induced cardiomyopathy compared with conventional myocardial pacing, particularly in adult [1, 2]. In contrast, atrial pacing has historically received less attention, and the right atrial appendage (RAA) remains the default site for atrial lead implantation despite growing evidence of its nonphysiologic consequences [3].

RAA pacing is associated with prolonged P‐wave duration (PWD), interatrial conduction delay, atrioventricular dyssynchrony, and impaired left atrial mechanical function [4], all of which may contribute to atrial remodeling, atrial fibrillation, and adverse clinical outcomes [5, 6]. These effects appear to be related to the cumulative burden of atrial pacing and, by extrapolation, may be particularly relevant in patients with long life expectancy and anticipated lifelong pacing exposure.

Bachmann's bundle (BB) is the dominant interatrial conduction pathway under normal sinus rhythm. Targeting this pathway for atrial pacing represents a physiologically appealing alternative to conventional atrial pacing sites. However, experience with BB pacing remains limited in adults, and no pediatric cases have been reported so far.

We report, to our knowledge, the first pediatric case of BB pacing, combined with left bundle branch area pacing (LBBAP).

### 1.1 Case Report

A 9‐year‐old boy (body weight 24.3 kg, height 135 cm) with congenital complete heart block (CHB) secondary to maternal anti‐Ro antibodies was referred for pacemaker system revision. He presented with fetal bradycardia and subsequently underwent implantation of an epicardial dual chamber pacing system on Day 26 of life. At 8 months of age, he developed pacing‐induced cardiomyopathy, presenting with heart failure and characterized by left ventricular (LV) dyskinesia at the left basal lateral epicardial pacing site. He was managed with reduction of the upper tracking rate to 100 bpm, and LV systolic function gradually improved and normalized by 14 months of age. He was then challenged with full tracking of his own sinus rate without recurrence of heart failure and LV dysfunction.

By 9 years of age, the epicardial generator reached end‐of‐life. Pacemaker interrogation demonstrated a non‐negligible atrial pacing burden of approximately 20%, at a lower rate of 60 bpm on DDD pacing. His underlying sinus P wave had abnormal P mitrale morphology with a prolonged PWD of 122 ms. Given the patient's prior history of pacing‐induced cardiomyopathy, the anticipated risk of epicardial lead failure with somatic growth, the consensus was to implant a new transvenous dual‐chamber system employing physiologic pacing.

The procedure was performed under general anesthesia combined fluoroscopic, electrophysiologic and transesophageal echocardiographic (TEE) guidance. After ultrasound‐guided access of the left axillary vein and creation of a subpectoral pocket, LBBAP was performed first. TEE was utilized to measure the interventricular septum (6.34 mm) and guide penetration to the subendocardium without LV perforation. A Medtronic 3830 lead (69 cm) (Medtronic, Minneapolis, MN, USA) was implanted via a C315S4 guiding sheath (Medtronic, Minneapolis, MN, USA) and positioned at the mid‐ventricular septum. Successful left bundle branch capture was confirmed by the appearance of an R′ wave in lead V1 and a short V6 R‐wave peak time (V6RWPT) of 74 ms. The paced QRS duration was narrowed measuring 106 ms.

**Figure 1:**
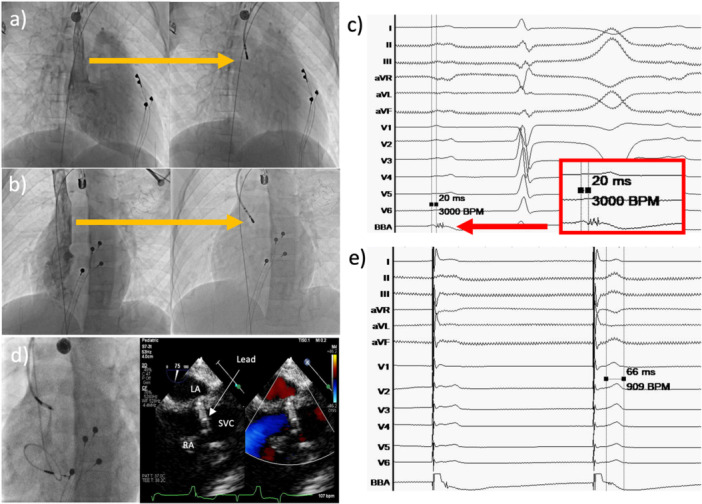
Electrophysiological mapping and pacing of Bachmann's bundle (BB). (a and b) Right (RAO) and left (LAO) anterior oblique fluoroscopic views showing contrast delineation of the superior vena cava–right atrial (SVC‐RA) junction. Mapping the high atrial septum using a 6Fr deflectable quadripolar catheter at the site with BB. (c) Intracardiac electrogram (EGM) recorded at the target site. The tracing identifies complex fractionated potentials (red arrow) timing well within the surface P‐wave, confirming the BB site prior to lead fixation. The magnified view (red box) highlights these characteristic low‐amplitude, fractionated BB potentials occurring approximately 20 ms after the onset of the surface P‐wave. (d) Fluoroscopy with contrast and TEE confirming the Medtronic 3830 lead tip position at the high atrial septum/SVC‐RA junction. (e) Paced ECG demonstrating successful electrical resynchronization. Note the isoelectric latency between the pacing stimulus and the onset of the P‐wave deflection. Pacing from the BB site significantly shortens the PWD to 66 ms compared to the intrinsic 122 ms.

To facilitate precise atrial substrate localization, a 6‐Fr deflectable quadripolar electrophysiology catheter (Livewire; Abbott, Abbott Park, IL, USA) was used for mapping. During mapping of the high atrial septum near superior vena cava‐right atrium (SVC‐RA) junction, low‐amplitude, multicomponent fractionated electrograms consistent with BB potentials were identified. These signals were timed approximately 20 ms after the onset of the surface P‐wave, distinct from the near‐field right atrial electrograms, confirming the electrophysiologic target prior to lead deployment (Figure 1a–c). Pre‐fixation TEE demonstrated an atrial septal thickness of 2.1 mm at the targeted site.

A second Medtronic 3830 lead (59 cm) was delivered via a C315S5 guiding sheath (Medtronic, Minneapolis, MN, USA) to the mapped BB region. The lead was screwed into the target site under fluoroscopic guidance. Good pacing and sensing thresholds could be achieved on the second attempt. However, lead dislodgement occurred during adjustment of atrial lead slack when clockwise torque and forward force were applied simultaneously, necessitating repositioning. On the third attempt, stable fixation was achieved, with capture of the interatrial conduction pathway confirmed by an isoelectric stimulus‐to–P‐wave onset and marked shortening of the paced P‐wave duration to 66 ms (Figure 1d,e). For all fixation processes, only approximately three clockwise rotations of the 3830 lead could be applied before significant back‐torque was encountered, limiting further advancement. In addition, atrial sensing and pacing thresholds were highly dependent on the final lead slack configuration, with only a limited range of configurations providing acceptable electrical parameters. After the delivery sheath was slit, there was a high pacing capture threshold requiring readjustment of atrial lead slack again. Postfixation TEE confirmed appropriate atrial lead position at the high atrial septum near the SVC–RA junction and adjacent to the right pulmonary vein, without protrusion into the left atrium or evidence of pericardial effusion.

**Figure 2:**
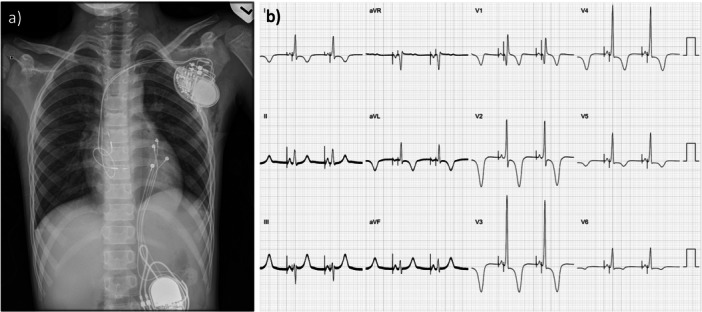
Final procedural outcome. (a) Postoperative CXR showing the transvenous dual‐chamber system. Note the U‐shaped lead slack in the RA. (b) Final DDD pacing ECG showing a narrow QRS with an rSR' pattern in V1 (confirming LBBAP) and a narrowed P‐wave morphology. Deep T‐wave inversions in V1–V4 were observed, consistent with cardiac memory commonly associated with LBBAP.

**Figure 3:**
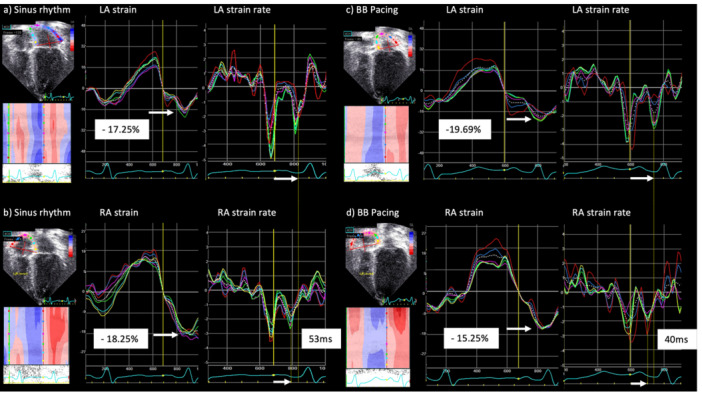
Assessment of mechanical atrial synchrony using speckle tracking echocardiography. Comparison of longitudinal strain and strain rate curves during intrinsic sinus rhythm (a and b) versus BB pacing (c and d). (a and b) The average longitudinal strain was −17.25% for the left atrium (LA) and −18.25% for the right atrium (RA). The interatrial conduction delay (difference in time to negative peak [TTNP] strain rate between LA and RA) was 53 ms. (c and d) BB pacing resulted in improved mechanical resynchronization. The interatrial conduction delay was reduced to 40 ms. LA and RA longitudinal strain were similar to sinus, measuring −19.65% and −15.25% respectively. GS, global strain; LA, left atrium; RA, right atrium; TTNP, time to negative peak.

The procedure time was 303 min and the total fluoroscopy dose was 366.28 cGy cm^2^. The final 12‐lead electrocardiogram (ECG) demonstrated physiologic activation, characterized by a narrow QRS complex with an rSR′ pattern in lead V1 and a markedly narrowed paced P‐wave morphology. Deep T‐wave inversions in V1–V4 were attributed to LBBAP‐associated cardiac memory (Figure 2). Serial postimplant device interrogations during early follow‐up demonstrated stable electrical parameters for both the BB and LBBAP leads. The atrial pacing threshold stabilized at 1.0 V at 0.4 ms. Although the atrial sensing amplitude initially decreased to 0.8 mV on postoperative Day 1, it demonstrated a favorable upward trend, reaching 2.6 mV by the end of the second week. A far‐field R‐wave (FFRW) of 0.6 mV was observed. Speckle‐tracking echocardiography confirmed mechanical atrial resynchronization. BB pacing reduced the interatrial conduction delay from 53 to 40 ms and normalized intraatrial activation patterns, while maintaining atrial longitudinal strain comparable to the patient's intrinsic conduction (Figure 3).

## 2 Discussion

Bi‐physiologic pacing, targeting atrial, and ventricular pathways, is feasible and safe in pediatric patients needing lifelong pacing, especially those requiring high atrial and ventricular pacing. It also highlights technical challenges of BB pacing in children.

### 2.1 Rationale for Bachmann's Bundle Pacing

Conventional RAA pacing is highly associated with adverse electrophysiologic effects—most notably, P‐wave prolongation and interatrial conduction delay—which are established independent predictors for the development of atrial fibrillation (AF) [4, 6, 7, 8]. As a physiologic alternative to RAA pacing, BB pacing has been developed to preserve interatrial synchrony. Lustgarten and colleagues described a novel, electrophysiology‐guided approach to BB pacing based on the identification of characteristic endocardial electrograms and confirmation via paced P‐wave morphology. This technique enables reproducible, anatomically targeted atrial conduction system pacing [9]. Unlike nonspecific atrial septal pacing, which yields inconsistent results [7], randomized adult data show that true BB pacing consistently preserves atrial synchrony and shortens paced PWD compared with RAA pacing, reducing the incidence and progression of AF compared with conventional RAA pacing [5, 10].

These considerations are particularly relevant for children requiring lifelong pacing. Although the immediate risk of atrial fibrillation is lower in children, Dai and colleagues recently demonstrated using high‐resolution epicardial mapping that the BB in a group of pediatric patients with congenital heart disease (CHD) already contains a considerable amount of intrinsic conduction disorders, low‐voltage areas, and complex potentials. Notably, this cohort predominantly consisted of patients with relatively mild forms of CHD, such as atrial or ventricular septal defects [11]. Consequently, physiologic BB pacing is pursued to mitigate the “cumulative burden” of iatrogenic dyssynchrony on a potentially vulnerable, growing heart. Furthermore, minimizing pacing‐related complications is critical given the cumulative clinical and economic burden of permanent pacing over a lifespan [12].

### 2.2 Technical Considerations of Bachmann's Bundle Pacing

Implementing atrial conduction system pacing in children presents unique challenges related to smaller vascular caliber, thinner septal tissue, and limited intracardiac working space, which increase the risk of vascular injury, cardiac perforation, and lead instability. Several technical considerations from this case merit emphasis.

First, preprocedural electrophysiologic mapping was essential to identify BB potentials and facilitate accurate positioning of the guiding sheath. Delivery sheaths currently available for BB pacing are designed primarily for adult anatomy and may not be optimally suited to the smaller atrial dimensions encountered in pediatric patients. In this context, mapping‐based localization of BB potentials served as a guide for sheath position adjustment which enabled precise targeting of the intended pacing site.

Second, atrial sensing and pacing performance were highly dependent on the final configuration of the atrial lead curve and lead slack. In this case, only a limited range of lead slack configurations provided acceptable sensing and pacing thresholds, reflecting the close spatial relationship between the lead tip and ring electrodes when positioned at the high atrial septum. This dependency appears more pronounced for BB pacing than for conventional RAA pacing, where lead geometry is less constrained by septal orientation. Therefore, difficulties could be encountered in adjusting the redundancy of lead slack to allow for somatic growth of pediatric patients. As the child grows, relative changes in the position of the atrial lead tip and ring electrode are anticipated, which may alter atrial sensing and pacing thresholds over time. The need to balance optimal electrical performance against sufficient lead slack to accommodate growth may therefore be challenging with BB pacing than with traditional atrial pacing sites. At present, long‐term outcome data for BB pacing in children are lacking, particularly regarding lead performance, sensing stability, and the impact of somatic growth. Continued follow‐up and broader pediatric experience are therefore required to determine the durability of this approach and to inform optimization of pacing tools for pediatric anatomy.

### 2.3 Safety of Bachmann's Bundle Pacing

While a large‐scale adult meta‐analysis has demonstrated no significant difference in overall perforation risk between atrial and ventricular electrodes, conventional lateral RAA placements are associated with an elevated risk of lead perforation [13]. Data regarding perforation risks specific to BB pacing in pediatrics is lacking. Conversely, targeting the BB region should theoretically decrease the risk of cardiac perforation compared to the RAA, given the relative thickness of the septal portion of the inferior superior vena cava [9]. However, data regarding perforation risks specific to BB pacing in pediatrics is lacking.

In our case, procedural safety was successfully enhanced through the use of multimodality imaging. Fluoroscopic guidance facilitated precise lead positioning, while TEE provided real‐time visualization of atrial septal anatomy, confirmation of lead depth, and immediate exclusion of left atrial perforation or pericardial effusion. The use of TEE is particularly valuable in pediatric patients, in whom the atrial septum is thin and fluoroscopic landmarks alone may be insufficient to ensure safe lead deployment. As demonstrated in this case, stable fixation of the atrial lead was achieved with only a limited number of clockwise rotations, underscoring the importance of direct visualization to confirm appropriate lead engagement and avoid excessive penetration.

## 3 Conclusion

To our knowledge, this is the first reported pediatric case of BB pacing combined with LBBAP in a child with congenital CHB requiring lifelong ventricular pacing, frequent atrial pacing, and abnormal P‐wave morphology. It shows that electrophysiology‐guided BB pacing is feasible and can be safely achieved in pediatric patients using multimodality imaging. While long‐term data are lacking, this supports further study of BB pacing in selected children with high pacing burden or pacing‐induced ventricular dysfunction.

## Consent

Written informed consent was obtained from the patient's parents for the publication of this case report and any accompanying images.

## Conflicts of Interest

The authors declare no conflicts of interest.
